# Carriage of CTX-M type extended spectrum β-lactamases (ESBLs) in gulls across Europe

**DOI:** 10.1186/s13028-015-0166-3

**Published:** 2015-11-02

**Authors:** Johan Stedt, Jonas Bonnedahl, Jorge Hernandez, Jonas Waldenström, Barry J. McMahon, Conny Tolf, Björn Olsen, Mirva Drobni

**Affiliations:** Centre for Ecology and Evolution in Microbial Model Systems, School of Natural Sciences, Linnaeus University, 391 82 Kalmar, Sweden; Department of Infectious Diseases, Kalmar County Hospital, 391 85 Kalmar, Sweden; Section of Clinical Microbiology and Infectious Diseases, Department of Medical Sciences, Uppsala University, 751 85 Uppsala, Sweden; UCD School of Agriculture and Food Science, University College Dublin, Belfield, Dublin 4, Ireland; Department of Laboratory Medicine, Clinical Microbiology, Östersund Hospital, 831 83 Östersund, Sweden

**Keywords:** ESBL, CTX-M, Wildlife, Birds, Gulls, Antibiotic resistance, *E. coli*, Europe

## Abstract

**Background:**

Extended spectrum β-lactamases (ESBLs), a group of enzymes conferring resistance to third generation cephalosporins have rapidly increased in *Enterobacteriacae* and pose a major challenge to human health care. Resistant isolates are common in domestic animals and clinical settings, but prevalence and genotype distribution varies on a geographical scale. Although ESBL genes are frequently detected in bacteria isolated from wildlife samples, ESBL dissemination of resistant bacteria to the environment is largely unknown. To address this, we used three closely related gull species as a model system and collected more than 3000 faecal samples during breeding times in nine European countries. Samples were screened for ESBL-producing bacteria, which were characterized to the level of ESBL genotype groups (SHV, TEM), or specific genotypes (CTX-M).

**Results:**

ESBL-producing bacteria were frequently detected in gulls (906 of 3158 samples, 28.7 %), with significant variation in prevalence rates between countries. Highest levels were found in Spain (74.8 %), The Netherlands (37.8 %) and England (27.1 %). Denmark and Poland represented the other extreme with no, or very few positive samples. Genotyping of CTX-M isolates identified 13 different variants, with *bla*_CTX-M-1_ and *bla*_CTX-M-14_ as the most frequently detected. In samples from England, Spain and Portugal, *bla*_CTX-M-14_ dominated, while in the rest of the sampled countries *bla*_CTX-M-1_ (except Sweden where *bla*_CTX-M-15_ was dominant) was the most frequently detected genotype, a pattern similar to what is known from studies of human materials.

**Conclusions:**

CTX-M type ESBLs are common in the faecal microbiota from gulls across Europe. The gull ESBL genotype distribution was in large similar to published datasets from human and food-production animals in Europe. The data suggests that the environmental dissemination of ESBL is high from anthropogenic sources, and widespread occurrence of resistant bacteria in common migratory bird species utilizing urban and agricultural areas suggests that antibiotic resistance genes may also be spread through birds.

## Background

Nosocomial infections (in human clinics) with bacteria harboring extended spectrum beta-lactamase (ESBL) genes started to appear in Europe in the mid-1980s, and has since then constituted an increasing everyday challenge in European clinics [1, 2]. A shift in the prevalence and genotypes of ESBLs in Europe occurred around 2000, when the CTX-M type ESBLs became the dominating class, with much greater penetration into the *Escherichia coli* populations than TEM or SHV type ESBLs, and is now found in human outpatient settings [3]. Today more than 170 different CTX-M genotypes are described (Bush K, Palzkill T, Jacoby G. Lahey Clinic. http://www.lahey.org/studies/, accessed September 2015), broadly divided in five groups (CTX-M group 1, 2, 8, 9 and group 25 [4]). In humans the most common CTX-M variants are *bla*_CTX-M-14_ and *bla*_CTX-M-15_ [3]. In food production animals ESBL-producing bacteria mainly occur in poultry, but are also reported with lower prevalence rates in cattle and swine, with *bla*_*CTX*-M-1_, *bla*_CTX-M-14_, *bla*_TEM-52_ and *bla*_SHV-12_ as the currently most frequently reported genotypes [5]. By comparing population genetic relationships between *E. coli* from humans and poultry it has been shown that antibiotic-resistant *E. coli* isolates are more frequently related than antibiotic-susceptible isolates are [5]. Further, a coherence between poultry and human ESBLs has been shown by sequence typed *E. coli* comparisons [6], illustrating transmission of resistant strains between sources. Consequently, both the clonality of *E. coli* and specific resistance genotypes are important study topics for fully understanding how transfer of resistance may occur. This is also concluded by Lazarus et al. in a systematic review, finding that food production animals, more apparent for poultry, may constitute a source of human extraintestinal ESCR-EC (expanded-spectrum cephalosporin-resistant *Escherichia coli*) infections. [7].

Recently, the occurrence of ESBL-producing bacteria in the environment has started to receive more attention and it has repeatedly been shown that they are regularly found in both wildlife and the environment [8–12]. The level of ESBL-producing bacteria in the environment seems to be higher in areas with high human densities, but are also found in seemingly remote areas, including *Escherichia coli* of O25b-ST131 clone harboring *bla*_CTX-M-15_ in gulls sampled at the isolated Commander Islands in Russia and 37 % ESBL harboring isolates in gulls sampled in Barrow, Alaska [13, 14]. Thus, wildlife has been pointed out as a potential reservoir for resistant bacteria [9, 10], and especially species that live close to humans [15–17]. In recent years, gulls (*Laridae*) have become particularly studied since they have many characteristics which make them suitable for resistance dissemination studies [11, 12, 18, 19]. Several gull species have large breeding distributions, are common in man-made environments, and to a large extent feed on human and food-animal waste. High carriage rates of ESBL-producing bacteria in gulls have been reported, for instance in a study from France where 9.4 % of the sampled Yellow-legged gulls (*Larus michaelis*) harbored ESBL-producing bacteria [11], and two studies of gulls in Portugal where 19.3 and 32 % of the gulls, respectively, carried ESBL-producing bacteria [19, 20]. A problem when addressing resistance occurrence in wildlife bacteria is that studies have generally been small, or not utilized standardized methodologies [10]. In order to gain a broader view of the resistance situation in the environment we undertook a standardized study where >3000 fecal samples from gulls in nine European countries were screened for ESBL-producing bacteria, with primary focus on prevalence of CTX-M genotypes. Specifically, we wanted to describe the occurrence and spatial variation in ESBL prevalence and *bla*_CTX-M_ genotype distribution in Europe. If the occurrence of ESBL is primarily driven by transfer of resistant genotypes from humans and food-production animals into wildlife, we hypothesize that ESBL prevalence and genotype distributions would conform to patterns seen in bacterial populations from these sources.

## Methods

### Studied avian species, sampling sites and sampling methodology

**Fig. 1:**
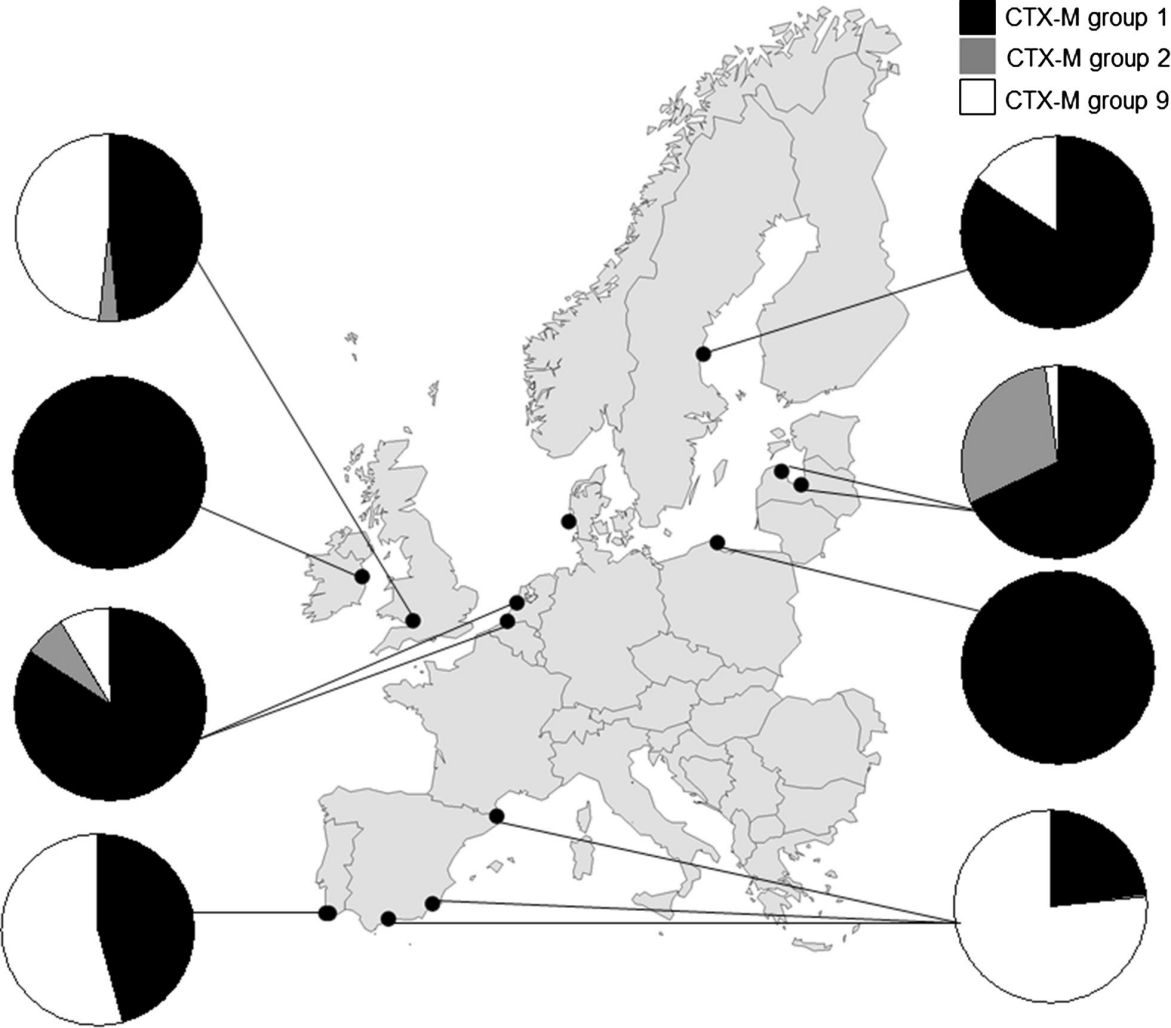
Distribution of CTX-M groups in *E. coli* from gulls in the nine European countries. In Spain (Emporda) one sample included a CTX-M group 8 (*bla*_CTX-M-8_) harboring isolate

Fecal samples were collected from Herring gulls (*Larus argentatus*) and Lesser-Black backed gulls (*Larus fuscus*) in northern Europe, and Yellow-legged gulls (*Larus michaelis*) in southern Europe (Table 1; Fig. 1). Field sampling was conducted from mid-June to early July 2009 in nine European countries. From each of the 14 sampling sites, 101–323 samples with bacterial growth were used (sample viability ensured by growth on non-selective media). Samples were collected from adult and subadult birds in or around breeding colonies. By collecting the samples during the late breeding season we limited the risk of sampling non-stationary birds. The sampling areas were chosen to be as similar as possible according to human activities, but different human density, suitable sampling locations, etc. made it impossible to have completely identical sites. To avoid that multiple samples were collected from one bird, samples were collected from separated flocks, and with fewer samples than individuals present at each site.

**Table 1:** Description of sampling sites and gull species

Country	Location	Species^a^	Samples (n)^b^	Description
Denmark	Blavand	*Larus fuscus* (60 %)	158	Coastline close to Roskilde
		*Larus argentatus* (40 %)		
England	Bristol	*Larus argentatus*	133	Central Bristol
Ireland	Howth	*Larus argentatus*	266	Suburb of Dublin close to fishing habour
Latvia	Daugava	*Larus argentatus*	323	Close to central Riga
	Kaltene	*Larus argentatus*	101	Countryside 60 km SV Riga
Netherlands	Borsele	*Larus argentatus*	280	Industry habour
	Rotterdam	*Larus fuscus* (90 %)	280	Industry habour
		*Larus argentatus* (10 %)		
Poland	Wladyslawowo	*Larus argentatus*	280	Coastline close to Gdansk
Portugal	Lagos	*Larus michaellis*	314	Fish market in Lagos
	Portimao	*Larus michaellis*	111	Coastline close to Portimao
Spain	Emporda	*Larus michaellis*	199	Citydump outside Emporda
	Mazarron	*Larus michaellis*	321	Breeding colonie close to Mazarron
	Malaga	*Larus michaellis*	175	Harbour in Malaga
Sweden	Hudiksvall	*Larus argentatus*	217	Archipelago outside Hudiksvall

^a^Percentage (%) represents the frequency of a gull species in the sampled flocks when there was more than one species present in the flock

^b^Number of viable samples from each sampling site

Sterile cotton wool swabs were swirled in freshly deposited fecal material on the ground where gulls had recently been roosting. Swabs were put in tubes containing bacterial freeze medium [Luria broth; BD, Sparks, USA, phosphate buffered saline containing 0.45 % Na-citrate, 0.1 % MgSO_4_, 1 % (NH_4_)_2_SO_4_, and 4.4 % glycerol] and immediately frozen in liquid nitrogen until arrival at the laboratory in Sweden where they were subsequently stored in −80 °C.

### Isolation of ESBL-producing bacteria

In total, 3158 fecal samples with bacterial growth (101–323 samples/sampling site) were available for analysis (Table 1). All samples were enriched in brain heart infusion broth (Becton–Dickinson, Franklin Lakes, NJ, USA), supplemented with 16 mg/L vancomycin, for 18–24 h in 37 °C, and subsequently inoculated on a ChromID™ ESBL plates (bioMérieux, Solna, Sweden), according to the manufacturer’s instructions. Presumptive ESBL producing colonies were isolated and species identity was confirmed by biochemical testing (standard fermentation analysis such as lactose ferm., ONPG, Voges-Proskauer, spot indole etc.) and MALDI/TOF (Bruker Daltonik GmbH, Bremen Germany). ESBL plates with bacterial growth of more than one bacterial species each presumable species was isolated. ESBL-production was confirmed with the cefpodoxime/cefpodoxime + clavulanic acid double-disc test (MAST Diagnostics, Bootle, UK). Samples were regarded as ESBL producing and further analyzed when zone diameter around cefpodoxime was ≥5 mm than the zone diameter around cefpodoxime + clavulanic acid, according to manufacturer’s instructions (MAST Diagnostics, Bootle, UK).

### Genetic determination of ESBL variants

The presence of *bla*_CTX-M_ genotype was detected using a previously described multiplex real-time PCR protocol [21], allowing designation of CTX-M subgroups (CTX-M group 1, 2, 9 and 8/25) [21] using StepOnePlus™ real-time PCR system (Applied biosystems). Positive isolates were sequenced using specific primers and protocols described previously [22, 23]. Sequencing was performed by Eurofins Genomics (Ebersberg, Germany).

The presence of *bla*_TEM_ and *bla*_SHV_ was detected using previously described primers [24] and a SYBR Green-based real-time PCR protocol [12].

## Results

### Prevalence of ESBL producing bacteria

ESBL producing bacteria were detected in 906 (28.7 %) of the 3158 samples. Since 44 samples contained more than one ESBL-producing bacterial species, the total number of ESBL producing bacteria was 950 (Table 2). The vast majority of the isolated ESBL were *E. coli* (902 isolates, 94.9 %) followed by 42 *Klebisella (41* *K. pneumoniae* and one *K. oxytoca)* and only six isolates from other bacterial genera: *Acinetobacter* spp 1 (*bla*_TEM_,), *Citrobacter* spp 2 (*bla*_TEM+_*bla*_SHV_), *Enterobacter* spp 2 (*bla*_SHV_) and *Proteus* spp 1 (*bla*_CTX-M-2_).

**Table 2:** Description of ESBL harboring samples and *bla*_CTX-M_ variants found in investigated countries

CTX-M group		Denmark	England	Ireland	Latvia	Poland	Portugal	Spain	Sweden	Netherlands
	Samples (n)	158	133	266	424	280	425	695	217	560
	ESBL genotypes (n)^a^	0	50	12	98	2	74	572	74	280
	ESBL samples(%)^b^	(0)	(27.1)	(4.5)	(17.4)	(0.7)	(12.7)	(74.8)	(20.7)	(37.8)
1										
	CTX-M-1		5.3^c^	2.6	4.2	0.4	1.4	3.2	2.3	21.3
	CTX-M-3		0.8		0.2			0.1		0.2
	CTX-M-15		4.5	0.4	3.5		0.5	1.3	13.8	2.1
	CTX-M-32			0.4	0.5	0.4	0.7	2.9		0.7
	CTX-M-55				0.2					
2										
	CTX-M-2		1.5		3.3					2.1
8										
	CTX-M-8							0.1		
9										
	CTX-M-9						0.2	2.7		0.2
	CTX-M-14		8.3				2.4	16.8	0.9	1.4
	CTX-M-14b		1.5		0.2			1.2	0.9	0.4
	CTX-M-24								1.4	
	CTX-M-27		0.8							
	CTX-M-65									0.2
	TEM^d^		3.0		0.7		6.6	26.2		3.6
	SHV^d^		12.0	1.1	10.1		5.6	27.8	14.7	17.9

^a^Total number of ESBL genotypes (n) in each country. Note that some bacterial strains carried more than one ESBL genotype

^b^Percentage (%) of total number of samples carrying ESBL

^c^Percentage (%) of the total n the total number of ESBL in each country umber of ESBL in each country belonging to a certain genotype

^d^Percentage (%) of TEM and SHV of the total number of ESBL in each country. However, note that a *bla*_TEM_ and *bla*_SHV_ genotype present in the same bacterial strain as another ESBL genotype could be *bla*_TEM-1_, *bla*_TEM-2_, or *bla*_SHV-1_ which are not considered true ESBL genotypes, but the original genes from which later genotype variants are derived from

The levels of ESBL positive samples varied significantly between countries. In Spain 74.8 % of the total number of samples carried ESBL. Also, The Netherlands, England and Sweden had high levels of ESBL producing bacteria (37.8, 27.1 %, respectively 20.7 %), while only 0.8 % of the samples in Poland, and in Denmark no ESBL positive samples were detected at all (Table 2).

### Distribution of ESBL variants

#### CTX-M

In total, 602 (66.4 %) of the ESBL harboring isolates carried CTX-M type ESBL. The number of detected *bla*_CTX-M_ variants was 13, distributed on five CTX-M group 1 variants, one variant of CTX-M group 2, one variant of CTX-M group 8/25, and six variants of CTX-M group 9 (Table 2). Group 1 (52.9 % of the total number of isolated CTX-M) and group 9 (42.2 %) dominated, and only a few isolates were designated group 2 (4.7 %) and group 8/25 (0.2 %) (Table 2). The most common genotypes were *bla*_CTX-M-1_ and *bla*_CTX-M-14_, but there was a large variation between *bla*_CTX-M_ variants between countries (Table 2). In Spain, Portugal and England *bla*_CTX-M-14_ was the most common CTX-M genotype. In The Netherlands there was a large dominance of *bla*_CTX-M-1_ (76.6 % of all CTX-M) while in Sweden *bla*_CTX-M-15_ (71.2 % of all CTX-M) was the most common. The CTX-M group 2 (*bla*_CTX-M-2_) was only found in three countries, Latvia, The Netherlands and England, and corresponded to 32.5, 7.8 and 3.4 % of the total number of CTX-M type ESBL, respectively. In Spain, one *E. coli* carried a *bla*_CTX-M-8_ while no sample carried genotypes from CTX-M group 25. A number of ESBL positive samples carried two different *bla*_CTX-M_ genotypes and a great part of those that carried *bla*_CTX-M_ were also positive for both *bla*_TEM_ and/or *bla*_SHV_ (data not shown).

#### TEM and SHV

ESBL positive isolates were screened for *bla*_TEM_ and *bla*_SHV_ genotypes, but not sequenced for specific genotype variants. In total 222 isolates were *bla*_TEM_ positive and 372 *bla*_SHV_ positive. Of these, 216 (80 %) *bla*_TEM_ and 242 (65 %) *bla*_SHV_ were present in the same isolate as another ESBL genotype. In those isolates it is not possible to exclude the commonly appearing variants *bla*_TEM-1_, *bla*_TEM-2_ and *bla*_SHV-1_ which are not true ESBLs. Thus, the level of true ESBLs could be lower for these variants.

## Discussion

Having investigated several of the European countries, particularly in the western parts, we can report a wide difference in ESBL prevalence, starting with none or very low ESBL prevalence in Denmark (0 %), Poland (0.7 %) and Ireland (4.5 %), to intermediate in Portugal (12.7 %), Latvia (17.4 %) and Sweden (20.7 %), and increasingly higher in England (27.1 %), The Netherlands (37.8 %), and Spain (74.8 %). Compared to current human clinical or veterinary data, most countries investigated show a high level of acquired ESBL resistance. Despite that on a national level prevalence rates could vary considerably, to our knowledge, the rates of ESBL producing bacteria reported in this study are greater than any previous study of clinically relevant bacteria from a wildlife source.

Standardized European studies covering prevalence of ESBLs in *E. coli* among community isolates from healthy humans are limited and not always including genotype details [25]. For European coverage EARS-net has a yearly report presenting levels of bacteria with resistance to third generation cephalosporins from clinical isolates [26]. In our study all countries except Denmark and Poland had ESBL levels higher then presented for clinical *E. coli* isolates from Europe in the corresponding countries, although AmpC was included in the Ears-net report and also the sample populations are very different. In countries such as Sweden and Spain, the levels of ESBL positive samples were considerably higher than reported from samples of healthy humans in separate studies in corresponding country [27–29]. Denmark and Poland were the only two countries that had lower prevalence in comparison to the data presented on EARS-net. From Denmark more recent data from 2011 presented by the DANMAPS yearly report show low but increasing levels of ESBLs in clinical samples and ESBLs were found in almost 8 % of blood culture samples from hospitalized humans [30]. Also in Portugal the levels of ESBL from our gull samples were low in comparison to the neighboring country Spain and a previously performed study on gulls from Portugal where ESBL was found in 32 % of the sampled gulls [19].

Spain showed extremely high levels of ESBLs, and in Sweden which is known to have low level of ESBLs in humans [31], 20.7 % of the gulls carried ESBL. This is much higher than previously found in gull studies performed in Sweden [12, 32]. The Netherlands have in coherence with Sweden low antibiotic usage in human clinical settings, but in gulls the levels were second highest when the studied countries are compared (37.8 %) [33]. However, The Netherlands have far higher antibiotic consumption in food-production [34].

Food-production animals are suggested to be an important source in the environmental dissemination of resistant bacteria, and the total consumption of antibiotics is much higher than what is used by human medicine [35]. The most comprehensive data for antibiotic resistance levels in food-production animals are presented for poultry, pigs and cattle in a yearly report by the European Food Safety Authority (EFSA). Compared to our study ESBL levels from seven of the countries are included (Denmark, England, Ireland, Latvia, Netherlands, Spain and Sweden). The levels of third generation cephalosporins resistance from 2008 were very low (<1 %) in cattle and pigs for all included countries. In poultry the levels of resistant *E. coli* varied between 0 and 26 % [36]. The overall trends corresponded, with highest levels of ESBLs in *E. coli* found in Spain from poultry and from our sampled gulls. Further ESBL was lacking in Danish poultry as well as the gull samples. Although trends should be the same, resistance levels may differ due to the randomized sampling approach used in the EFSA material.

In our gull material significant differences in genotype distributions could be seen between countries. In Spain, Portugal and England *bla*_CTX-M-14_ was dominant, while *bla*_CTX-M-1_ and *bla*_CTX-M-15_ was most frequently detected in the other surveyed countries (Table 2). This is in coherence with the human situation, where *bla*_CTX-M-14_ followed by *bla*_CTX-M-15_ are the most common genotypes in humans while *bla*_CTX-M-1_ and *bla*_CTX-M-14_ are most frequently detected in domestic animals [36, 37]. The pattern seen in gull isolates is to large extent in coherence with the pattern of *bla*_CTX-M_ genotype distribution in human isolates in Europe [3]. Livermore et al. [3] have elegantly summarized CTX-M data from humans in Europe in a review article and here *bla*_CTX-M-14_ is noted as one of the most frequent genotypes in Spain which is in coherence with results. Further, a study on *E. coli* isolated at Spanish hospitals showed high similarity in genotype frequency with our results (52 % of all CTX-M was *bla*_CTX-M-9_ and 39 % *bla*_CTX-M-14_) [38]. Also in Portugal and England there was a dominance of *bla*_CTX-M-14_ in isolates from gulls which is not the situation in human isolates. Separate publications from humans in UK and Portugal, and also a previous study on gulls in Portugal, found that *bla*_CTX-M-14_ was scarce in these [19, 39, 40]. This contrast could be explained by sampling site deviation from the “general picture”, or possibly dissemination from other sources as for example food-production animals. Studies show that *bla*_CTX-M-14_ genotype is frequent in Spanish and Portuguese poultry [41–43].

In England, Ireland and Sweden, included in our study, Livermore et al. [3] reports *bla*_CTX-M-15_ as the most frequently occurring genotype in isolates from humans and *bla*_CTX-M-1_ is also frequently occurring. This is in coherence with our results where *bla*_CTX-M-1_ was dominant in each country except Sweden which showed a large dominance of *bla*_CTX-M-15_. Interestingly, *bla*_CTX-M-15_, which is the dominating variant in humans in many parts of Europe, was not common in samples from gulls, except in those sampled in Sweden. This is another example of overlapping patterns between observations in wild birds and in humans, as *bla*_CTX-M-15_ is also the most common CTX-M variant in humans in Sweden [3, 44].

In contrast, The Netherlands where the human antibiotic consumption in humans are comparable to Sweden but far higher in food-production animals there was a total dominance of *bla*_CTX-M-1_ [34]. This is considered as a CTX-M genotype most often found in poultry and other food-production animals in Europe [45, 46] and the poultry production in The Netherlands is the most intense in Europe [47]. High similarities between ESBL variants in humans and poultry in the Netherlands have been seen with a dominance of *bla*_CTX-M-1_ [6].

The CTX-M group 2 is unevenly distributed in Europe, with different variants mainly spread in Russia and Eastern Europe [2]. This pattern was seen in the gull material where 32 % of the CTX-M isolated from *E. coli* in Latvia belonged to *bla*_CTX-M-2_. It is also noteworthy that one *bla*_CTX-M-8_ was isolated from a gull *E. coli* in Spain, to our knowledge the first record of *bla*_CTX-M-8_ from wildlife [9].

## Conclusions

Despite low availability of easily comparable data from human and veterinary settings, levels of ESBL are seemingly higher in wild gulls in some regions of Europe. Certainly, the diet of gulls make them exposed to a variety of bacteria from different sources. Gulls are gregarious, especially during breeding, which could contribute to rapid spread of ESBL between individuals in a local population. Spread between gulls could also be mediated by the environment, as shown for *Salmonella* which can be maintained in a breeding colony of gulls between breeding seasons [48]. Since many gull species migrate, sometimes even between continents, there is a risk that these birds will contribute to the global spread of antibiotic resistance genes. The results from this study are therefore remarkable and the high environmental ESBL levels, as seen in gull fecal samples, alarming.
